# Supplementary material for: Functional Characterization of Allelic Variations of Human Cytochrome P450 2C8 (V181I, I244V, I331T, and L361F)
Source: Int J Mol Sci. 2023 Apr 28;24(9):8032. doi: 10.3390/ijms24098032 (PMC10178350; doi:10.3390/ijms24098032)
Supplement: Supplementary file 1 [file ijms-24-08032-s001.zip › ijms-2339937-supplementary.pdf]

SUPPORTING INFORMATION

Functional Characterization of Allelic Variations of Human Cytochrome P450 2C8 (V181I, I244V, I331T, and L361F)

Yoo-bin Lee, Vitchan Kim, Sung-Gyu Lee, Gyu-Hyeong Lee, Changmin Kim, Eunseo Jeong,

and Donghak Kim.

TABLE OF CONTENTS

Figure S1. Amino acids sequence alignment of P450 2B1 and P450 2C8

Figure S2. The constructed docking model of I244V mutation.

Figure S3. The mutation locations of V181I and I331T.

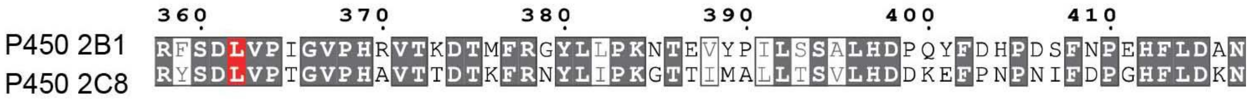

Figure S1. Amino acids sequence alignment of P450 2B1 and P450 2C8. The multiple sequence alignment results as produced by T-coffee. The Leu362 of P450 2B1 and the Leu361 of P450 2C8 are indicated with red color.

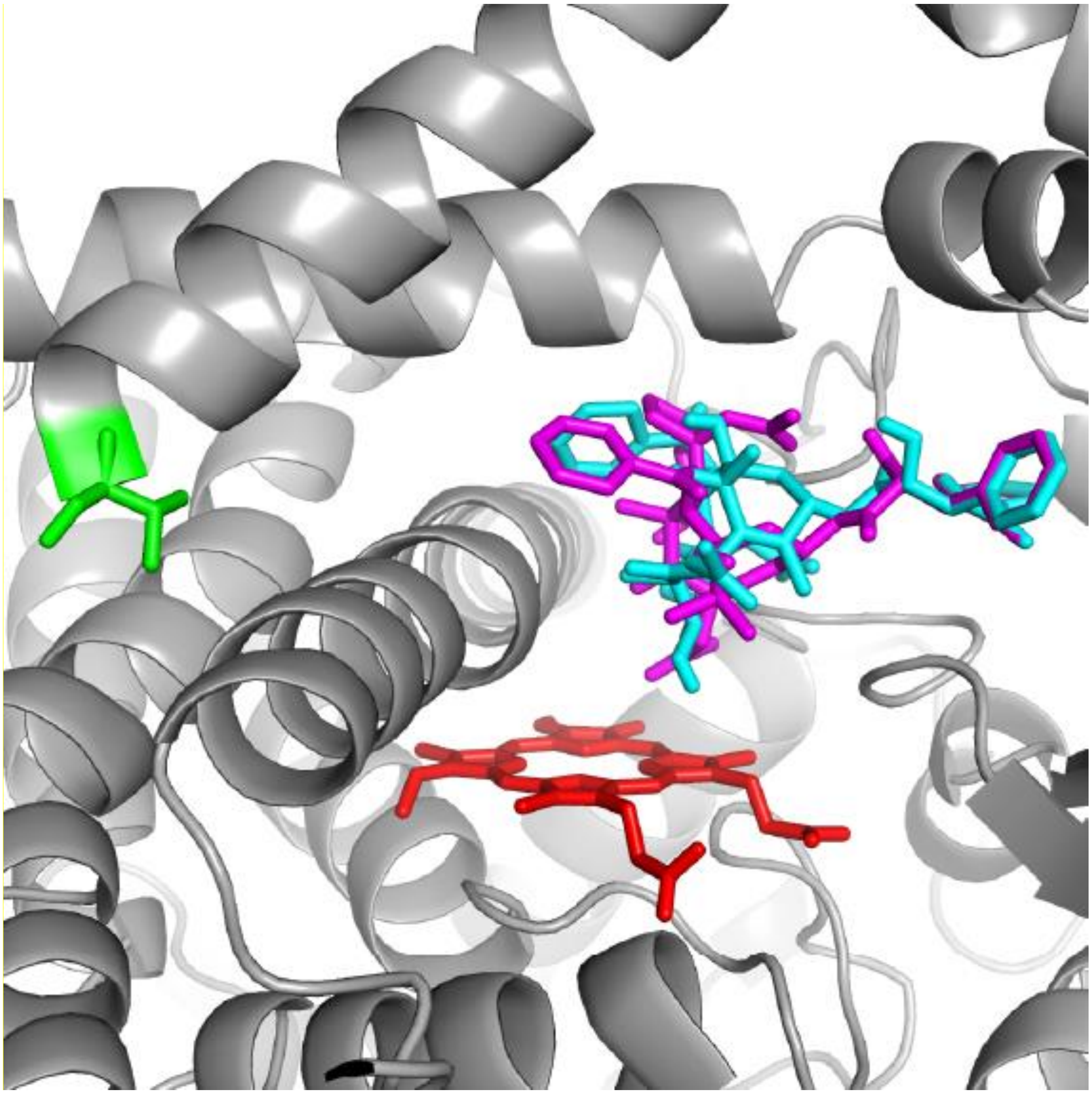

Figure S2. The constructed docking model of I244V mutation. The molecular docking models were constructed using the reported X-ray crystal structure of P450 2C8 (pdb ID; 1PQ2). Paclitaxel docking model of P450 2C8 wild type (cyan) and I244V mutation (magentas) have similar position and orientation at the active site access channel.

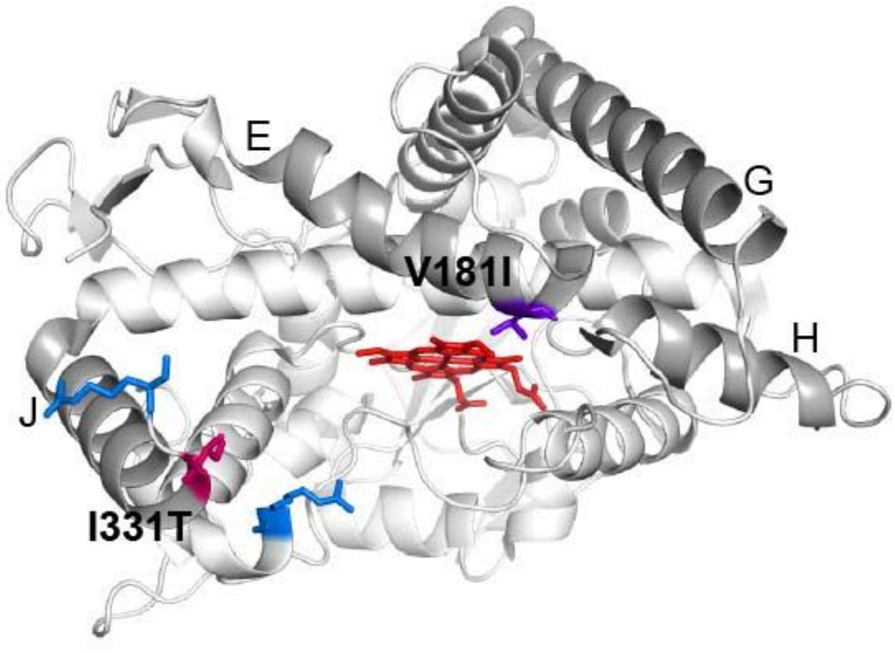

Figure S3. The mutation locations of V181I and I331T. The mutation of V181I (purple) was located at the C-terminus of helix E of P450 2C8 structure and the I331T mutation (red) was located at the C-terminus of J-helix. The basic Arg residues are indicated in blue.
